# Sex differences in glucagon-like peptide-1 signalling and the implications for Alzheimer’s disease: A narrative review

**DOI:** 10.1017/S1092852926101291

**Published:** 2026-10-29

**Authors:** Serene Lee, Esha Purewal, Natalie Rasgon, Angela Kwan, Christine E. Dri, Roger Ho, Roger S. McIntyre

**Affiliations:** 1 Brain and Cognition Discovery Foundation, Canada; 2 Queen’s University, Canada; 3 Stanford University, USA; 4 Brain and Cognition Discovery Foundation, University Health Network, Canada; 5 National University of Singapore Yong Loo Lin School of Medicine, Singapore; 6 Department of Psychiatry, University of Toronto, Canada

**Keywords:** Alzheimer’s disease, GLP-1, T2DM, sex, metabolism, insulin

## Abstract

Alzheimer’s disease (AD) is a progressive neurodegenerative disorder and is the most common form of dementia. Women are differentially affected by AD, and several sex-specific mechanisms have been explored, including hormonal differences (eg, estrogen). Glucagon-like peptide-1 (GLP-1R) are located in brain regions implicated in the pathogenesis of AD and shows preliminary evidence of modulating sex-specific pathways that may underlie differential disease risk and progression. Herein, we synthesize the extant literature on the mechanisms of GLP-1 in neurobiological systems relevant to AD pathology. Metabolic signalling (eg, GLP-1, insulin) intersects with known sex-specific pathways hypothesized to subserve AD progression in women. The preceding effects may be attributable to the disruption of estrogen signalling during the postmenopausal period, which impairs hippocampal insulin receptor function and exacerbates mitochondrial dysfunction. In addition, these mechanisms provide an explanation for AD comorbidities (eg, type 2 diabetes mellitus [T2DM]). The multidimensional effects demonstrated in GLP-1 paradigms provide a compelling rationale for further investigation of the role of metabolic signalling on sex-specific alterations in AD. Evidence across preclinical and early clinical studies suggests that GLP-1RAs may differentially influence neurobiological pathways implicated in AD pathogenesis, particularly those that interact with hormonal and metabolic systems.

## Introduction

Alzheimer’s disease (AD) is a progressive neurodegenerative disorder characterized by cognitive impairments and memory loss.^1^ The hallmark pathophysiological features of AD are the presence of neuritic plaques and neurofibrillary tangles (NFTs), resulting from amyloid-beta peptide (Aβ) accumulation in the brain (eg, medial temporal lobe, neocortical structures) and hyperphosphorylated tau proteins, respectively.^1^ ^,^^2^ In AD, women are differentially affected insofar as two-thirds of persons using health services for AD are women, and they consistently face higher rates of premature mortality relative to men.^3^ ^,^^4^

Additionally, women are often diagnosed with more severe AD pathology, characterized by greater tau protein accumulation and markedly noticeable NFTs.^5^ Sex dimorphisms have a cardinal role in the pathogenesis, progression, age of onset, and treatment response in AD.^6^ The prevalence of dementia varies by gender, with approximately 8.1% of women and 5.4% of men over the age of 65 affected, nearly 70% of whom have AD.^7^ In addition to differences in prevalence, soluble and insoluble Aβ levels have been reported to be higher in female than male mice, suggesting sex-specific differences in the pathological burden.^8^

Although biological mechanisms play a central role in these differences, alternative explanations have been suggested. One hypothesis is survivor bias, where men may be at greater risk of cardiovascular mortality before reaching the age at which AD typically manifests.^3^ Another hypothesis involves historical disparities in educational attainment among women, given that education is an established protective factor against AD.^3^ Existing literature has examined estrogens as a key biological factor underlying sex differences in AD among women, but few studies have explored other molecular and cellular mechanisms.

Insulin is a peptide hormone that plays a role in memory and cognition, as well as in neuronal development and plasticity.^6^ Moreover, type 2 diabetes mellitus (T2DM) is a well-known risk factor for AD; for example, incretin deficits are observed in both conditions.^9^ ^–^^12^ Glucagon-like peptide-1 receptor agonists (GLP-1RAs) are Food and Drug Administration (FDA)-approved incretin mimetics for T2DM and obesity, but are also able to cross the blood–brain barrier (BBB) and exert effects on central nervous system (CNS) GLP-1 receptors, which are involved in neuroprotective and neuroplasticity-enhancing effects.^13^ ^–^^15^

In addition, the presence of GLP-1 receptors in brain regions implicated in the pathogenesis of AD (eg, the hippocampus) suggests they may modulate inflammation and oxidative stress.^14^ ^,^^16^ In keeping with this view, a testable hypothesis is that sex differences in GLP-1 receptor signalling in the brain contribute to the increased development and/or progression of AD in females. Moreover, notwithstanding these differences, an overwhelming majority of the literature focuses on male AD outcomes. Herein, we synthesize the extant literature on sex differences in GLP-1 signalling that may play a role in gender-based AD outcomes, and provide an update on estrogen-mediated pathways.

## Methods

**Figure 1.**
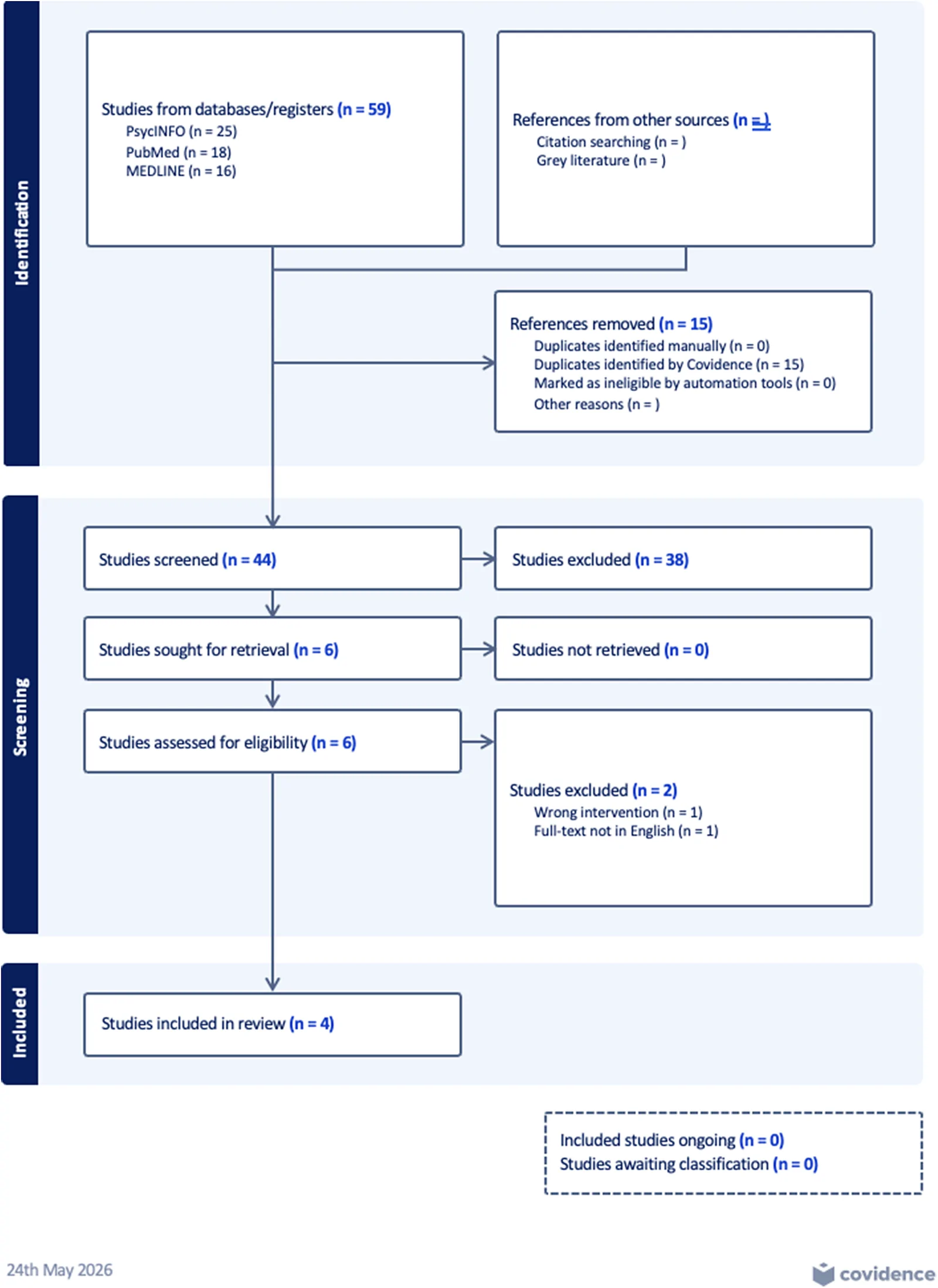
PRISMA flow diagram. Figure 1. Long description.

A comprehensive literature search was conducted across PubMed, Ovid MEDLINE, PsycINFO, and Google Scholar from database inception to May 24, 2026 (Figure 1). Additional relevant articles were identified through manual searches of the reference lists of retrieved publications. The following Boolean search strategy was used: ([“glucagon-like peptide-1”] OR [“GLP-1”]) AND (“Alzheimer’s disease”) AND ([sex] OR [gender]). The review was limited to articles published in English. Studies were included if they examined GLP-1 signalling or GLP-1 receptor agonists in relation to AD and reported sex-stratified outcomes or investigated biological mechanisms relevant to sex differences. Relevant studies identified through manual reference searching that examined broader dementia outcomes were also considered if they addressed the review objective. Studies that did not report sex-specific analyses or investigate biological mechanisms relevant to sex differences were excluded from the review (Table 1).

**Table 1.** Eligibility Criteria Table 1. Long description.

Inclusion criteria
1. A primary or post-hoc analysis,
2. Clinical or preclinical study,
3. At least one outcome of the investigation must be the implication of GLP–1 in sex-related differences for AD,
4. Population of interest must have modeled AD or AD,
5. Comorbidities (eg, obesity, type 2 diabetes mellitus).
Exclusion criteria
1. Full text unavailable online,
2. Publication language not in English

## Results

A total of four studies were identified from the search (Table 2). Abdulhameed et al. investigated the effects of incretin receptor agonists (IRAs) targeting either or both the GLP-1R or glucose-dependent insulinotropic polypeptide receptor (GIPR) in APP/PS1 mouse models displaying AD characteristics.^8^ These researchers administered either exenatide, dulaglutide, semaglutide, DA4-JC, or DA5-CH intravenously and found significant sex differences in IRA uptake at the 60-minute mark.^8^ DA4-JC uptake was higher in females than males in all brain regions studied, excluding the striatum and hypothalamus, which showed equivalent uptake.^8^ Abdulhameed et al. were unclear on what drove the sex differences, but they postulated an increase in GLP-1R expression in female brains compared to male brains, as evidenced by a new transcriptomics tool visualizing GLP-1R mRNA.^8^

**Table 2.** Descriptive Characteristics of Included Studies Table 2. Long description.

Author(s)	Subjects	Subject criteria	Intervention	Duration	Intervention outcome	Significance
Abdulhameed et al.^8^	Male and female CD–1 mice (8–10 weeks old)	APP/PS1 mouse model displaying elevated Aβ plaque load, neuronal loss within the neocortex and hippocampus, and early deficits in learning and memory	IRAs targeting either or both GLP–1R or GIPR administered intravenously (exenatide, dulaglutide, semaglutide, DA4-JC, and DA5-CH)	Collection of blood occurred at 5, 15, 30, and 60 min time points after administration	Significant sex differences in IRA uptake at the 60 min mark were most common with DA4-JC. Its uptake was higher in females than males in all the brain regions studied except the striatum and hypothalamus, which showed equivalent uptake	Unclear what is driving the sex differences. Information about sex differences in GLP–1 and GIP expression in the brain is limited. A new transcriptomics tool visualizing GLP–1R mRNA expression in the brain between men and women aged 20–70 years shows that later in life, women have greater expression than men
Cas et al.^18^	Female participants (*n* = 16) and male participants (*n* = 16)	AD diagnosis	Slow-release exenatide (2 mg subcutaneously once a week) or no treatment	32 weeks	The primary endpoint was the change in ADAS-Cog11 cognitive test score at 32 weeks versus baseline. A gender interaction with treatment was observed (*p* = 0.04), due to worsening of the ADAS-Cog11 score in women randomized to exenatide (*p* = 0.018)	Some evidence suggests that GLP–1-RA may exert sex-related effects
Duarte et al.^17^	10-month-old WT (control) and 3xTg-AD female mice	Genetic model for AD (3xTg-AD) that develops an age-related progressive neuropathological phenotype	Subcutaneous injection with liraglutide (0.2 mg/kg) or saline injection (0.9% sterile NaCl)	28 days	Levels of brain estradiol and GLP–1 were increased in female mice with early AD-like pathology compared to WT ones (for brain GLP–1 levels: *F*(2,13) = 2.686; *p* = 0.106; for brain estradiol levels: *Z* = −2.191, *p* = 0.030 for 3xTg-AD versus WT mice)	Liraglutide treatment tended to normalize both estradiol and GLP–1 levels (for brain estradiol levels: *Z* = −1.358, *p* = 0.222 for 3xTg-AD + Lira versus WT mice; *Z* = −0.548, *p* = 0.662 for 3xTg-AD + Lira versus 3xTg-AD mice
Sun et al.^19^	Retrospective cohort study using data from a global health research network (*n* = 87 229)	Patients with T2DM initiating GLP–1RAs or metformin as first-line monotherapy were included	Dementia incidence	2004–2024	Subgroup analyses showed consistent benefit across age and sex, with the strongest effect among older adults and females. Both sexes benefited, although females had a slightly lower aHR (0.83; 95% CI 0.79–0.89) compared with males (0.90; 95% CI 0.84–0.97). A modest but significant reduction in VaD risk was observed exclusively in female patients treated with GLP–1 RAs (aHR 0.88; 95% CI 0.79–0.99), whereas no significant differences were noted in the overall population	These findings may be influenced by estrogen’s vascular and neuroprotective effects, which could enhance the benefits of GLP–1RAs or metformin in postmenopausal women

Similarly, Duarte et al. performed subcutaneous injections of liraglutide in female genetic mouse models for AD that developed an age-related progressive neuropathological phenotype.^17^ In the female mice with early AD-like pathology compared to the WT female mice, the levels of brain estradiol (*p* = 0.03) and GLP-1 (*p* = 0.106) were shown to increase, suggesting that the administration of liraglutide treatment partially normalized both estradiol and GLP-1 levels.^17^ Liraglutide decreased the cortical amyloid-beta levels, reduced astrocyte- and microglia-mediated inflammation, and promoted neurogenesis and proliferation.^17^ The researchers suggest that this study supports the use of GLP-1 mimetics to mitigate the earlier AD-like pathological features in mature females.^17^

Cas et al. administered exenatide subcutaneously in participants with AD, with the primary endpoint being a change in the ADAS-Cog11 cognitive test.^18^ A gender interaction with treatment was observed (*p* = 0.04) due to worsening of the ADAS-Cog11 score in women randomized to exenatide (*p* = 0.018), suggesting that some GLP-1RAs may exert sex-related effects.^18^ These researchers state that these results should be interpreted in the context of AD, which displays several sex-related features.^18^ Their data are consistent with the possible effects of estrogens on brain structure and function, including learning and memory.^18^ Weight parameters may suggest that many pharmacodynamics and pharmacokinetics factors contribute to sex-related disparities in the efficacy of anti-obesity drugs.^18^

Sun et al. conducted a retrospective cohort study examining GLP-1RA or metformin monotherapy and subsequent dementia incidence.^19^ Subgroup analyses showed consistent benefit across age and sex.^19^ However, the confidence intervals overlapped, and no formal interaction test was reported.^19^ Both sexes benefited, although females had a slightly lower aHR (0.83; 95% CI 0.79–0.89) compared with males (0.90; 95% CI 0.84–0.97).^19^ A modest but significant reduction in VaD risk was observed exclusively in female patients treated with GLP-1RAs (aHR 0.88; 95% CI 0.79–0.99), whereas no significant differences were noted in the overall population.^19^ These researchers suggest that the effects may be subserved by estrogen’s vascular and neuroprotective effects, which could enhance the benefit of GLP-1RAs in postmenopausal women.^19^ In addition, sex-specific metabolic and inflammatory differences in diabetes provide the impetus for the potential role of biological variables in modulating treatment efficacy.

## Discussion

Herein, through a narrative review, we identified evidence from the current literature supporting structural and molecular differences in GLP-1, insulin, and metabolic signalling that may be responsible for the increased susceptibility of women to the incidence and severity of AD. Estrogen has previously been identified as a key protective agent against AD molecular changes (eg, NFTs, Aβ).^5^ ^–^^7^ We have shown here that estrogen may exert these effects, at least partly, by regulating metabolic states in the body. In addition, we postulate that the postmenopausal drop in estrogen, which impairs mitochondrial homeostasis, decreases insulin’s ability to exert neuroprotective effects, thereby predisposing to conditions known to increase AD risk (eg, obesity).^3^ ^,^^7^ ^,^^20^

The findings of protective effects of GLP-1 signalling, which are disrupted in the postmenopausal female brain, support the hypothesis that sex-specific alterations in incretin-mediated metabolic regulation may constitute a critical mechanistic link between estrogen decline and heightened vulnerability to neurodegenerative processes characteristic of AD. Specifically, impaired GLP-1 receptor activity in the absence of estrogenic support may exacerbate mitochondrial dysfunction, reduce insulin sensitivity within central neural circuits, and potentiate amyloidogenic and tauopathic pathways, thereby amplifying disease severity in women.^3^ ^,^^7^ ^,^^20^ Mechanistic findings from the studies examined also suggest a greater expression of GLP-1 mRNA in the female brain than in the male brain. However, mechanistic uncertainty, study heterogeneity, and limited sex-specific evidence warrant caution when extending these findings to AD prevention.

Several limitations should be considered when interpreting the summarized findings. The overarching limitation is that the extant literature examining GLP-1 signalling in the context of sex differences and AD is limited in scope, with few studies directly investigating sex-stratified outcomes. Much of the mechanistic evidence is derived from preclinical mouse models, which may not fully capture the complexity of human neurodegenerative processes or hormonal transitions such as menopause. In the preclinical literature, including studies of GLP-1RAs, male animals predominate in experimental designs. This underrepresentation of female models restricts the interpretation of sex-specific mechanisms. Pharmacodynamic findings may vary as GLP-1RA penetration into specific brain regions can differ by sex, compound characteristics, and age. Significant heterogeneity exists across the study designs, animal strains, age groups, and outcome measures. Further heterogeneity across study designs, animal and human populations, GLP-1RA agents, routes of administration, age groups, and outcome measures limits direct comparability across findings. Hence, there is a need for large, controlled, sex-stratified clinical trials and mechanistic studies.

## Data Availability

The datasets generated and/or analyzed during the current study are available in the PubMed repository, https://pubmed.ncbi.nlm.nih.gov/, OVID repository, https://ovidsp.ovid.com/, and PsycINFO repository, https://www.apa.org/pubs/databases/psycinfo

## Long descriptions

### Figure 1. Long description

The flowchart is divided into three vertical sections: Identification, Screening, and Included.

1. Identification Phase:

- Top-left box: Studies from databases/registers (n = 59), including PsycINFO (n = 25), PubMed (n = 18), and MEDLINE (n = 16).

- Top-right box: References from other sources (n = blank), including Citation searching and Grey literature.

- An arrow leads to a side box: References removed (n = 15), consisting of 0 manual duplicates, 15 duplicates identified by Covidence, and 0 marked as ineligible by automation tools.

2. Screening Phase:

- Studies screened (n = 44) leads to Studies excluded (n = 38).

- Studies sought for retrieval (n = 6) leads to Studies not retrieved (n = 0).

- Studies assessed for eligibility (n = 6) leads to a side box: Studies excluded (n = 2) due to Wrong intervention (n = 1) and Full-text not in English (n = 1).

3. Included Phase:

- Studies included in review (n = 4).

- A separate dashed box at the bottom right notes: Included studies ongoing (n = 0) and Studies awaiting classification (n = 0).

Navigate back to Figure 1..

### Table 1. Long description

The table is divided into two main sections.

Inclusion criteria:

1. A primary or post-hoc analysis.

2. Clinical or preclinical study.

3. At least one outcome of the investigation must be the implication of G L P 1 in sex-related differences for A D.

4. Population of interest must have modeled A D or A D.

5. Comorbidities such as obesity or type 2 diabetes mellitus.

Exclusion criteria:

1. Full text unavailable online.

2. Publication language not in English.

Navigate back to Table 1..

### Table 2. Long description

The table consists of seven columns: Author(s), Subjects, Subject criteria, Intervention, Duration, Intervention outcome, and Significance.

* Row 1: Abdulhameed et al. studied male and female C D 1 mice (8 to 10 weeks old) with A P P / P S 1 mutations. Interventions included I R As targeting G L P 1 R or G I P R (exenatide, dulaglutide, semaglutide, D A 4 J C, and D A 5 C H). Blood was collected at 5, 15, 30, and 60 minute intervals. Results showed significant sex differences in I R A uptake at 60 minutes, particularly with D A 4 J C, which was higher in females across most brain regions.

* Row 2: Cas et al. studied 16 female and 16 male human participants with A D diagnosis. Intervention was slow-release exenatide (2 milligrams once a week) for 32 weeks. A gender interaction was observed (p equals 0.04) due to worsening A D A S Cog 11 scores specifically in women randomized to exenatide.

* Row 3: Duarte et al. studied 10 month old female W T and 3 x T g A D mice. Intervention was subcutaneous liraglutide (0.2 milligrams per kilogram) for 28 days. Brain estradiol and G L P 1 levels were higher in A D mice than W T; liraglutide treatment tended to normalize these levels.

* Row 4: Sun et al. conducted a retrospective cohort study of 87,229 patients with T 2 D M from 2004 to 2024. They found G L P 1 R As reduced dementia risk across both sexes, with a stronger effect in females (a H R 0.83) than males (a H R 0.90). A significant reduction in V a D risk was observed exclusively in female patients.

Navigate back to Table 2..
